# Inhibition of AKT with the Orally Active Allosteric AKT Inhibitor, MK-2206, Sensitizes Endometrial Cancer Cells to Progestin

**DOI:** 10.1371/journal.pone.0041593

**Published:** 2012-07-24

**Authors:** Alok Pant, Irene I. Lee, Zhenxiao Lu, Bo R. Rueda, Julian Schink, J. Julie Kim

**Affiliations:** 1 Division of Gynecologic Oncology, Department of Obstetrics and Gynecology, Northwestern University, Chicago, Illinois, United States of America; 2 Division of Reproductive Biology Research, Department of Obstetrics and Gynecology, Northwestern University, Chicago, Illinois, United States of America; 3 Vincent Center for Reproductive Biology, Department of Obstetrics and Gynecology, Massachusetts General Hospital/Harvard Medical School, Boston, Massachusetts, United States of America; Baylor college of Medicine, United States of America

## Abstract

Progestin resistance is a major obstacle to treating early stage, well-differentiated endometrial cancer as well as recurrent endometrial cancer. The mechanism behind the suboptimal response to progestin is not well understood. The *PTEN* tumor suppressor gene is frequently mutated in type I endometrial cancers and this mutation results in hyperactivation of the PI3K/AKT pathway. We hypothesized that increased activation of AKT promotes an inadequate response to progestins in endometrial cancer cells. Ishikawa cells stably transfected with progesterone receptor B (PRB23 cells) were treated with the AKT inhibitor, MK-2206, which effectively decreased levels of p(Ser473)-AKT in a dose-dependent (10 nM to 1 uM) and time-dependent manner (0.5 h to 24 h). MK-2206 inhibited levels of p(Thr308)-AKT and a downstream target, p(Thr246)-PRAS40, but did not change levels of p(Thr202/Tyr204)ERK or p(Thr13/Tyr185)SAPK/JNK, demonstrating specificity of MK-2206 for AKT. Additionally, MK-2206 treatment of PRB23 cells resulted in a significant increase in levels of progesterone receptor B (PRB) protein. Microarray analysis of PRB23 cells identified PDK4 as the most highly upregulated gene among 70 upregulated genes in response to R5020. Inhibition of AKT further upregulated progestin-mediated expression of PDK4 but did not affect another progestin-responsive gene, SGK1. Treatment of PRB23 cells with R5020 and MK-2206 independently decreased viability of cells while the combination of R5020 and MK-2206 caused the greatest decrease in cell viability. Furthermore, mice with xenografted tumors treated with MK-2206 alone or with progesterone alone exhibited modest reductions in their tumor volume. The largest decrease in tumor size was observed in the mice treated with both MK-2206 and progesterone; these tumors exhibited the least proliferation (Ki67) and the most apoptosis (cleaved caspase-3) of all the treatment groups. In summary, inhibition of AKT stabilizes the Progesterone Receptor B and augments progesterone response in endometrial cancer cells that have hyperactivated AKT.

## Introduction

Endometrial cancer is the most common gynecologic malignancy in the United States. In 2010, 43,470 new cases were anticipated resulting in 7950 deaths [1]. The vast majority of endometrial cancer cases are related to unopposed estrogen action are referred to as Type I endometrial cancers. The most common genetic mutation that occurs in approximately 50–80% of all cases in Type I endometrial cancer is in the tumor suppressor gene *PTEN* [2], [3]. A mutation in the *PTEN* gene results in downstream constitutive activation of the phosphatidylinositol 3′-kinase (PI3K)/AKT pathway [4]. AKT is a serine/threonine kinase that activates different pathways promoting cellular survival and inhibiting apoptosis [4], [5]. It has been previously demonstrated that there are increased levels of activated AKT in endometrial cancer and that this may portend a poor prognosis in these patients [6]. Additionally, it has been shown that inhibition of the AKT pathway in endometrial cancer cells results in increased apoptosis, thus showing that modulation of this pathway could play an important therapeutic role [7], [8].

Endometrial cancer is usually treated with surgical removal of the uterus and adjuvant therapy as indicated by the specific pathology. For early stage disease this treatment results in excellent 5-year survival rates of greater than 90% [9]. However, this definitive surgery precludes any further fertility. As obesity rates increase among the population, this results in increasing numbers of younger women with endometrial cancer and fertility-sparing therapy will become more invaluable. Currently, progestins are used as primary therapy in advanced or recurrent disease, in patients who are not operative candidates due to medical morbidity, and in patients hoping to preserve their future fertility. Multiple case series of patients treated with progestins have been published with encouraging, though not absolute, results. Response rates for endometrial hyperplasia with atypia have ranged from 67–82% and response rates for low grade endometrial cancer range from 50–70% (reviewed in [10]). For those patients who do not respond to progestin therapy, hysterectomy is recommended. This clinical situation is common as the percentage of patients younger than 45 diagnosed with endometrial cancer now ranges from 5 to 29% [10], .

Progesterone mediates its inhibitory effects on the endometrium and endometrial cancer via the progesterone receptor (PR), an intracellular steroid receptor with A and B isoforms. An increase in the response rates to progestin therapy and improved survival outcomes have been reported in tumors with a higher percentage of PR [12]. PRA lacks 164 amino acids from the N-terminus and both isoforms are translated from individual mRNA species of a single gene under the control of distinct promoters and are considered functionally distinct [13]. While the specific roles for PRA and PRB remain unclear in endometrial cancer, studies suggest that PRB may be the primary isoform responsible for the growth inhibitory and tumor suppressive actions of progesterone in vitro. In PRB-stably transfected Ishikawa cells, MPA treatment resulted in inhibition of proliferation, migration, and invasion, while PRA-stably transfected Ishikawa cells failed to inhibit these processes [14], [15]. Additionally, PRB-transfected Hec50co cells treated with progesterone demonstrated significant reductions in cell proliferation and invasion, whereas Hec50co cells expressing PRA had only modest inhibitory effects [16].

MK-2206 is an orally active, allosteric inhibitor of AKT. Numerous preclinical studies have demonstrated efficacy in inhibiting AKT and promoting cancer cell death as a single agent as well as in combination with other chemotherapeutic agents [17], [18], [19], [20]. Clinical trials are in progress using this novel compound for cancer therapy for various solid tumors. MK-2206 was shown to be generally well tolerated at doses up to 60 mg by mouth QOD and resulted in plasma concentrations within the accepted therapeutic range [21]. Currently, there is an open phase II trial sponsored by the NCI for patients with advanced or recurrent endometrial cancer. The primary outcomes are progression- free survival and objective tumor response.

In this study, we report that inhibition of the AKT pathway by MK-2206 results in decreased viability and increased apoptosis of endometrial cancer cells. In addition, inhibition of AKT increases PRB protein levels, modifies the expression of progestin-regulated genes, and synergizes with progestin to decrease tumor volume of xenografts. Combinatorial treatment with progestins and AKT inhibitors could be considered to sensitize endometrial adenocarcinoma to progestin therapy.

## Materials and Methods

### Ethics Statement

All animal experiments were approved by Northwestern University Animal Care Committee.

### Endometrial Cancer Cells

PRB23 Ishikawa cells were obtained from L. Blok (Erasmus University, Netherlands). Ishikawa cells originated from a well-differentiated endometrial adenocarcinoma with a known PTEN mutation [14]. The PRB23 cells were generated by stable transfection of the pcDNA3.1 PRB expression vector into Ishikawa cells devoid of endogenous PR [14]. The PRB23 cells were maintained in DMEM/F12 with 10% FBS, sodium pyruvate, penicillin/streptomycin, hygromycin, and G418. At approximately 60–80% confluence, the cells were serum starved overnight in DMEM/F12. The cells were then treated the next day with vehicle, 100 nM MK-2206 (2 h pretreatment), 10 nM R5020, or a combination of the MK-2206 and R5020. MK-2206 (Merck) was obtained through the Cancer Therapy Evaluation Program.

### Western Blot

Whole cell lysates were obtained on ice using the M-PER Mammalian lysis solution (Thermo Scientific) supplemented with protease and phosphatase inhibitors (Sigma). The concentration of protein in the lysates was measured using the Micro BCA kit (Thermo Scientific). Isolated protein samples were run on 8% acrylamide gels and then transferred onto polyvinylidene difluoride membranes (Whatman). Membranes were blocked in 5% Bovine Serum Albumin (BSA, Sigma) in TBS-T at room temperature for one hour. Membranes were then incubated overnight at 4°C with primary antibodies against phosphorylated (Ser473)-AKT, phosphorylated (Thr 308)-AKT, phosphorylated PRAS40, PRAS40, phosphorylated(Thr202/Tyr 204) ERK, ERK, (pThr183/Tyr185) SAPK/JNK, JNK, cleaved PARP, cleaved Caspase 3 (Cell Signaling), and PR (Dako). The blots were washed four times in TBS-T at room temperature and then incubated with secondary peroxidase-conjugated goat anti-rabbit or goat anti-mouse antibody for one hour at room temperature. The membranes were developed with the ECL Super Signal West Femto detection kit (Thermo Scientific). The membranes were stripped using Restore Western Blot Stripping Buffer (Pierce) and probed with an antibody to beta-actin (Sigma) for a loading control.

### Cell Viability

To assess for cell viability, the AlamarBlue Cell Viability Reagent (Invitrogen) was used. PRB23 cells were seeded in a 96-well clear bottom black plate at approximately 1,500 cells per well in a 37°C incubator. The cells were allowed to attach overnight and the next day, were washed with PBS and then serum starved overnight in serum-free DMEM/F12. The next day, the cells were pre-treated with 1 µM MK-2206 for one hour and then treated with 100 nM R5020 for 120 hours. At the end of the treatment time, 10 µL of AlamarBlue reagent was added to each well for two hours and then the fluorescence signal was measured on the Synergy HT plate reader from Bio-Tek with the KC4 3.4 software at 530/590 nm to determine cell viability.

### Microarray Analysis and Statistical Analysis

Three separate PRB-Ishikawa cell cultures were used for microarray analysis. The experimental conditions were vehicle or treatment with 10 nM R5020 for 2 h. All RNA samples were processed at the Genomics Core Facility in the Center for Genetic Medicine at Northwestern University (Chicago, IL). The quality of total RNA was evaluated using the Bioanalyzer 2100 (Agilent Technologies, Inc., Santa Clara, CA). 1.5 µg of each RNA sample, with 260/280 and 28S/18S ratio of greater than 1.8, was used to make double-stranded cDNA. Quality checks and probe level processing of the Illumina microarray data were further made with the R Bioconductor package, lumi (http://www.bioconductor.org/packages/release/bioc/html/lumi.html). Data processing also included a normalization procedure utilizing the quantile normalization method [22] to reduce the obscuring variation between microarrays, which might be introduced during the processes of sample preparation, manufacturing, fluorescence labeling, hybridization, and/or scanning. Hierarchical clustering and Principal Component Analysis were performed on the normalized signal data to assess the sample relationship and variability. Probes absent in all samples were filtered out according to Illumina’s detection p-values in the downstream analysis. Differential gene expression between the different conditions was assessed by a statistical linear model analysis using the bioconductor package limma, in which an empirical Bayes method was used to moderate the standard errors of the estimated log-fold changes of gene expression, and resulted in a more stable inference and improved power, especially for experiments with small numbers of microarrays ([23]; http://www.bioconductor.org/packages/release/bioc/html/limma.html). The moderated t-statistic p-values derived from the limma analysis above were further adjusted for multiple testing by Benjamini and Hochberg’s method to control false discovery rate (FDR). The lists of differentially expressed genes were obtained by the FDR criteria of <5% and fold change cutoff of >1.5, and visualized by volcano plots.

### Real Time PCR

RNA was isolated from cells using TRIzol reagent (Invitrogen) according to the manufacturer’s protocol. Concentration and purity of extracted RNA were determined using the ND-1000 Spectrophotometer (NanoDrop). Total RNA samples were DNase I (Zymo Research) treated to remove any contaminating DNA. Total RNA was reverse-transcribed with the Smart MMLV reverse transcriptase (Invitrogen) for the synthesis of cDNA using the manufacturer’s protocol. Real-time PCR was performed using specific primers (Applied Biosystems) to PDK4 and SGK1 and the housekeeping gene TBP. The fold change in expression was calculated using the ΔΔCt method [24], with TBP as an internal control. All PCR reactions were carried out on an ABI PRISM 7000 Sequence Detection System (Applied Biosystems) for 40 cycles (95°C for 15 s, 60°C for 1 min) after 10 min incubation at 95°C.

### Tumor Xenografts

Six-week old CD-1 nude, ovariectomized female mice were purchased from Charles River Laboratories. All animal experiments were approved by Northwestern University Animal Care Committee. One million PRB23 cells were suspended in 1∶2 PBS/Matrigel (BD Biosciences) in a total volume of 100 µL and subcutaneously injected into the right and left dorsum of each mouse. A total of 32 mice were injected in order to have 8 mice per treatment group. The tumors were allowed to grow for 8 weeks. Out of 32 mice, tumors grew in 28 mice. In addition, 2 mice died for unknown reasons. The remaining 26 mice were divided into 4 groups, vehicle (6), progesterone (7), MK-2206 (7), and MK-2206+progesterone (6). Progesterone pellets (50 mg, 21-day release for 2.4 mg/day) were implanted subcutaneously. The mice were given 120 mg/kg of MK-2206, 3 times per week for 9 days in a volume of 0.3 mL of 30% Captisol. All toxicity studies have been performed in rodents by Merck and 120 mg/kg is the dose recommended to study effects on tumors without causing toxicity [25]. The vehicle control group mice were given 0.3 mL of 30% Captisol. Mice were weighed before and after treatments. Tumor sizes were measured with calipers through the course of 8 weeks as well as after treatments. Tumor volumes were calculated using the formula:

*Tumor volume*  = 1/2(*length* × *width*)^2^ [25]. Tumors were excised and processed for immunohistochemistry.

### Immunohistochemistry

Tissues were fixed in formalin and paraffin embedded, and 4 µm tissue sections were placed on glass slides. The tumor sections were de-paraffinized. Heat induced epitope retrieval was then performed in a 10 nM sodium citrate buffer with 0.05% Tween (Sigma) at pH 6.0. The citrate buffer was pre-warmed in a water bath at 99°C and then the slides were placed in the buffer for 45 minutes. The slides were allowed to cool for 30 minutes at room temperature and were washed in TBS-T for 5 minutes. The Dako EnVision HRP IHC kit was used. 3% hydrogen peroxide was applied to the tissue sections for 10 minutes followed by 2 washes for 10 minutes in TBS-T. Protein block was applied for 30 minutes at room temperature. The tissue sections were then incubated with primary antibodies to PR (Dako) or cleaved Caspase 3 (Cell Signaling) overnight at 4°C in a humidified chamber. Anti-rabbit or anti-mouse secondary antibody was then applied to the tissue sections for 1 hour at room temperature and then washed in TBS-T twice for 5 minutes. DAB solution was applied to each tissue section for 5 minutes and then rinsed. Mayer’s Hematoxylin was used to counterstain and the slides were rinsed. The sections were then placed in a solution of 28% ammonium hydroxide and then rinsed. The sections were dehydrated via 2 changes of 95% ethanol, 2 changes of 100% ethanol, and 2 changes of Xylene. The sections were then mounted onto cover slips using Cytoseal XYL mounting media (Richard-Allan Scientific). The slides were then visualized using the Axiovert 200 (Zeiss) microscope and pictures taken with the Zeiss Axiocam camera.

## Results

### MK-2206 Inhibits AKT

**Figure 1:**
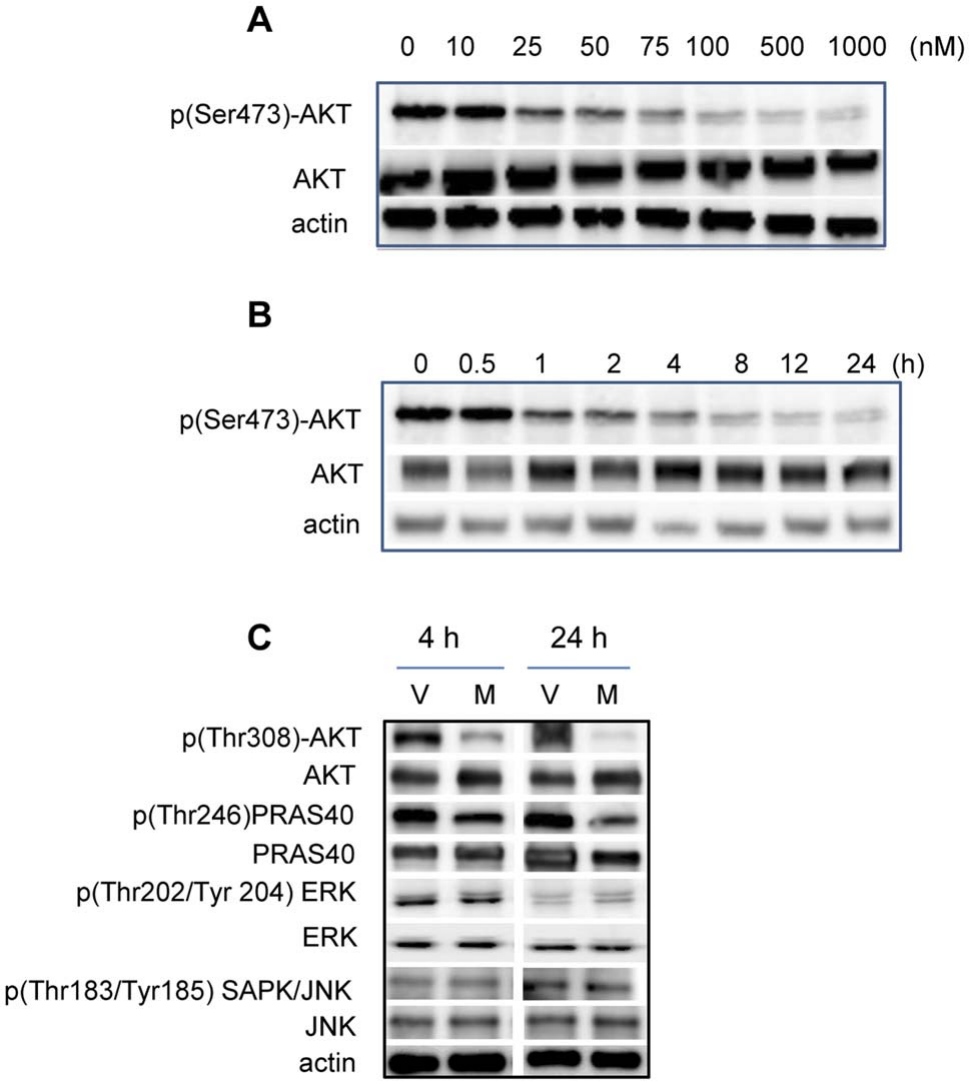
The AKT inhibitor, MK-2206 inhibits AKT. PRB-specific Ishikawa (PRB23) cells were treated with A) increasing concentrations (0–1 uM) of MK-2206 for 24 h and B) 100 nM MK-2206 for various times (0–24 h). Protein levels of p(Ser473)-AKT, AKT and actin were measured by Western blot. C) PRB23 cells were treated with 100 nM MK-2206 for 4 h or 24 h and levels of p(Thr308)-AKT, AKT, p(Thr246)PRAS40, PRAS40, p(Thr202/Tyr204) ERK, ERK, p(Thr183/Tyr185) SAPK/JNK, JNK and actin were measured by western blot.

It has previously been reported that Ishikawa cells carry a PTEN mutation and this results in constitutive downstream activation of AKT [7], [8]. In order to determine whether MK-2206 inhibited AKT in the PRB23 cells, cells were treated with increasing concentrations of MK-2206 (0–1 µM) and incubated for various times (0–24 h) (Fig. 1A, 1B). Phosphorylated (Ser473)-AKT was detected in cells treated with vehicle and levels progressively declined with increasing concentrations of MK-2206 (Fig. 1A). Total AKT levels were not affected by treatment with MK-2206. Levels of p(Ser473)-AKT decreased, as early as 1 h, and remained lower than vehicle treated cells at 24 h (Fig. 1B). Levels of p(Thr308)-AKT also declined with 100 nM MK-2206 treatment at both 4 h and 24 h (Fig. 1C). PRAS40 is a direct substrate of AKT and levels of p(Thr246)-PRAS40 decreased upon MK-2206 treatment at 4 h and 24 h, indicating that the AKT activity is decreased (Fig. 1C). Next, in order to demonstrate that MK-2206 was specific to the AKT pathway in PRB23 cells, levels of other phosphorylated kinases, which are not direct targets of AKT were measured. Levels of p(Thr202/Tyr204)-ERK as well as p(Thr183/Tyr185)-JNK did not change upon MK-2206 treatment either at 4 h or 24 h (Fig. 1C).

### Inhibition of AKT Increases PRB Protein Levels

**Figure 2:**
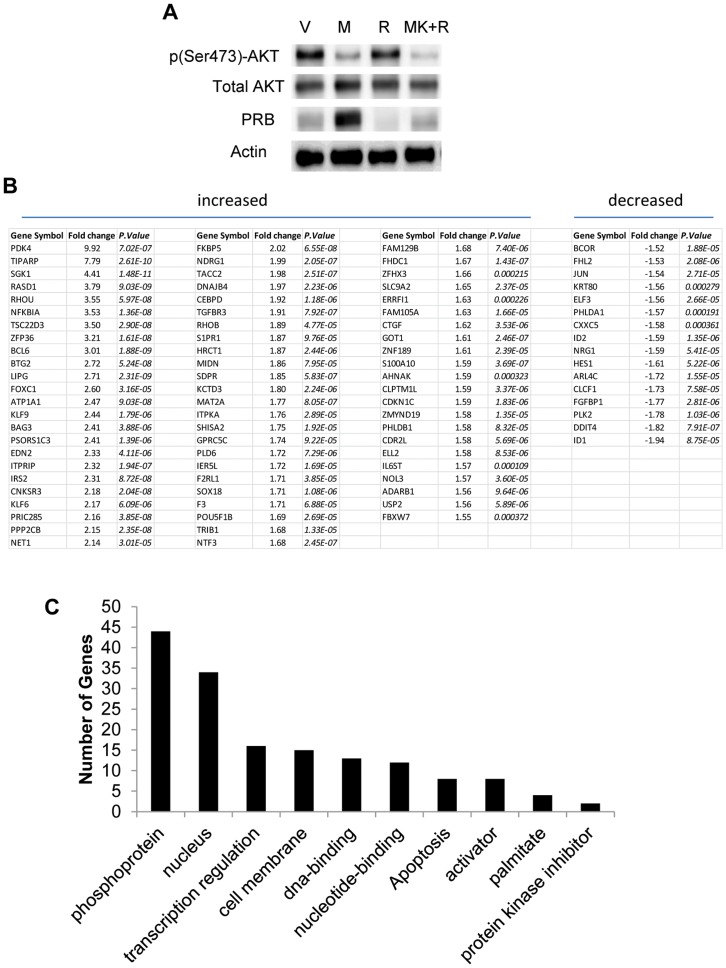
MK-2206 increases PRB protein. A) PRB-specific Ishikawa (PRB23) cells were treated with vehicle, 100 nM MK-2206, 10 nM R5020, or a combination of MK-2206+R5020 (M+R) for 4 h and protein levels of p(Ser473)-AKT, AKT, PRB, and actin were measured by Western blot. B) Microarray analysis of PRB23 cells treated with vehicle or 10 nM R5020 was performed and genes significantly regulated by 1.5-fold or greater were identified. C) Gene ontology categories analysis was done using DAVID for genes regulated by more than 1.5-fold by R5020.

Interestingly, treatment with MK-2206 also resulted in a significant increase in PRB levels in the PRB23 cells, indicating that AKT affects levels PR protein in these cells (Fig. 2A). Given the antagonistic function of progesterone in the endometrium, the implications of modulating PR levels are significant. First, a microarray was performed to identify PRB-regulated genes in PRB23 cells. Specifically, PRB23 cells were treated with 10 nM R5020 for 2 h and thus only the early response genes to progestins would be identified. A total of 70 genes were significantly upregulated by more than 1.5 fold and 16 genes were downregulated significantly (Fig. 2B). The most highly upregulated genes were PDK4 (9.92-fold), TIPARP (7.79-fold), and SGK1 (4.41-fold). Genes were further analyzed with the ‘functional annotation clustering’ tool of the Database for Annotation, Visualization and Integrated Discovery (DAVID) [26]. Genes were categorized according to the SP-PIR Keywords database. The category that included the highest number of genes that were regulated by R5020 was “phosphoproteins” and the second highest were genes that its products were associated with the nucleus (Fig. 2C).

**Figure 3:**
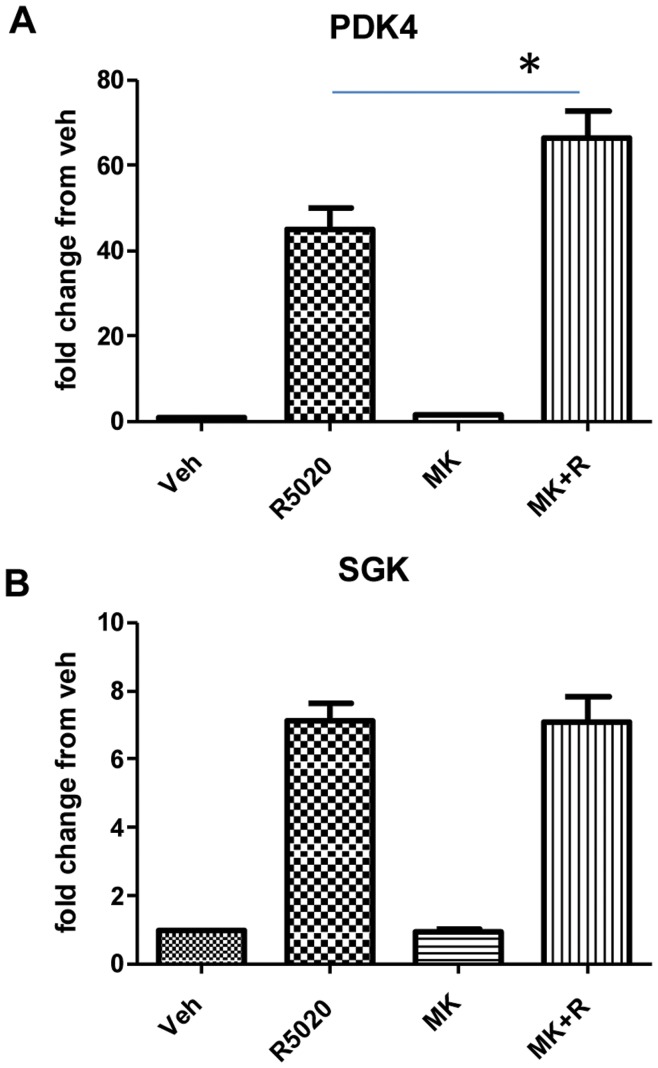
Influence of MK-2206 on R5020-regulated genes. PRB23 cells were treated with vehicle, 10 nM R5020, 100 nM MK-2206, or a combination of MK-2206+R5020 (M+R) and gene expression of PDK4 and SGK1 was measured using real-time PCR. Data are presented as fold changes from vehicle treated samples and represent the mean ± sem of 6 independent experiments. “*” denotes statistical significance compared to vehicle control. P≤0.05.

Next, the influence of AKT on PRB target genes was examined. PRB23 cells were treated with 10 nM R5020 in the presence or absence of 100 nM MK-2206. Expression of PDK4 and SGK1 were then measured by real time PCR. PDK4 and SGK1 were categorized as phosphoproteins. As depicted in Figure 3, both PDK4 and SGK1 were significantly upregulated by R5020, confirming the microarray data. MK-2206 treatment alone did not influence expression of any of the genes tested compared to vehicle-treated cells. When cells were treated in combination with R5020 and MK-2206, expression of PDK4 significantly increased, however, SGK1 expression did not change with the combination treatment compared to R5020 alone. This differential regulation of PRB target genes in the PRB23 cells when AKT is inhibited strongly suggests a more complex mechanism of regulating genes than simply increasing levels of PRB protein.

### MK-2206 and Progestin Decreases Cell Viability and Tumor Size

**Figure 4:**
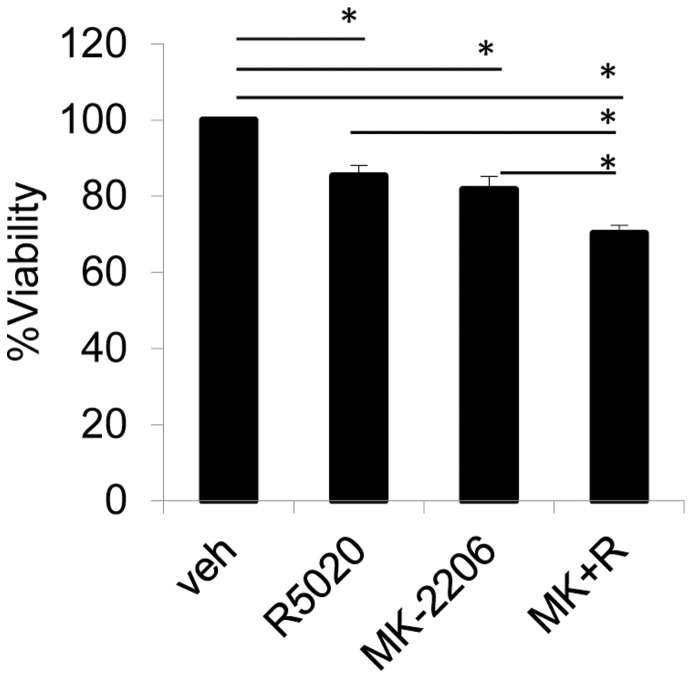
Combination of MK-2206 and R5020 significantly decreases cell viability. PRB23 cells were treated with vehicle, 100 nM R5020, 1 uM MK-2206, or a combination of MK-2206+R5020 (M+R) for 5 days and cell viability was measured using the Alamar Blue assay. Data are presented as % viability from vehicle treated samples and represent the mean ± sem of 4 independent experiments.

In order to measure the effects of AKT and progestin on cancer cell viability, PRB23 cells were treated with R5020 in the presence or absence of MK-2206 and cell viability was measured. While R5020 and MK-2206 independently decreased viability of cells over the course of 5 days the combination of R5020 and MK-2206 caused the greatest decrease in viability (Fig. 4).

**Figure 5:**
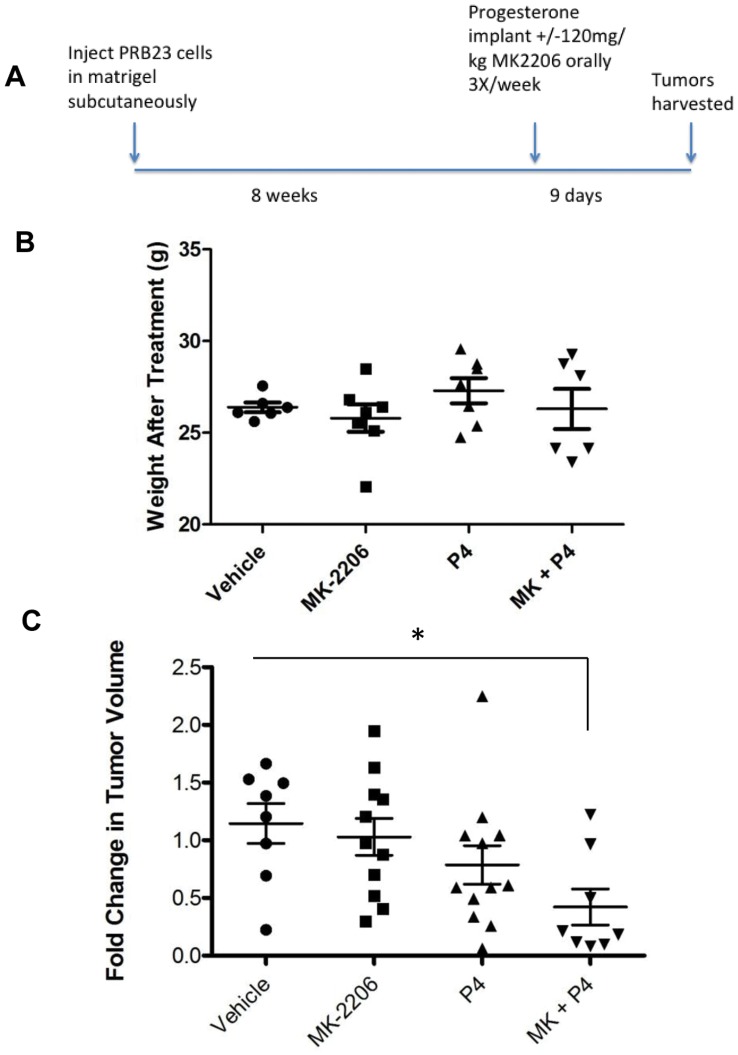
Combination of MK-2206 and R5020 promotes tumor regression. A) PRB23 cells were xenografted subcutaneously in CD1 nude mice. After 8 weeks, mice were treated with vehicle, 120 mg/kg MK-2206 orally, 3 times a week for 9 days, progesterone or combination of MK-2206 and progesterone. B) Weights of mice were measured before and after treatments and C) tumors were measured before and after treatments and volume was calculated. “*” denotes statistical significance compared to vehicle control. P≤0.05.

Next, the effects of progesterone and MK-2206 were tested in xenografted tumors that developed from subcutaneous injection of the PRB23 cells in nude mice. The tumors were grown for 8 weeks and subsequently treated for 9 days with vehicle, progesterone, MK-2206, or progesterone+MK-2206 (Fig. 5A). Weight of mice did not change significantly with the treatments (Fig. 5B). Compared to the mice treated with vehicle, the mice treated with MK-2206 alone or progesterone alone exhibited modest reductions in their tumor volume based on fold change from the tumor size before treatment. The largest decrease in tumor size was observed in the mice treated with both MK-2206 and progesterone (Fig. 5C).

**Figure 6:**
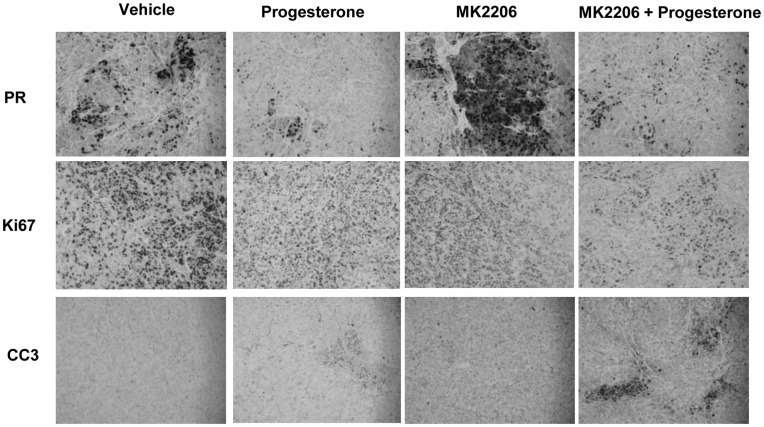
Combination of MK-2206 and R5020 decreases proliferation and increases apoptosis in tumors. Xenografted tumors were fixed, embedded and sectioned. Immunohistochemical staining of tumors for PR, Ki67 and cleaved casapse-3 (CC3) was performed.

Immunohistochemical analysis was done on tumor sections using antibodies to progesterone receptor, Ki67 and cleaved caspase-3 (Fig. 6). Staining for PR was evident in the tumors, although not all cells were positive for PR. Progesterone treatment decreased levels of PR both in the presence and absence of MK-2206. In contrast, MK-2206 treatment promoted an increase in PR protein levels in the tumors, similar to what was observed in endometrial cancer cells (Fig. 2A). Proliferation of the cells was measured by Ki67 protein levels, which decreased with progesterone, MK-2206 as well as the combination progesterone+MK-2206 treatment. The lowest levels of Ki67 staining was observed with the combined treatment, suggesting that the combined treatment more effectively decreases proliferation of the cancer cells. Levels of cleaved caspase-3, an indicator of apoptosis, were negligible in the tumors of vehicle treated mice, while staining was evident in the tumors of progesterone or MK-2206 treated mice. The combined treatment resulted in the highest expression of cleaved caspase-3, indicative of increased apoptosis.

## Discussion

In this study, we demonstrated that MK-2206 efficiently decreased levels of p(Ser473)-AKT and p(Thr308)-AKT in the PTEN-mutated Ishikawa cells. As a result, levels of p(Thr246)-PRAS40, a downstream target of AKT, decreased as well, indicative that the activity of AKT was inhibited by MK-2206. This inhibitor did not affect levels of other kinases, such as pERK and pJNK demonstrating specificity for this compound to the AKT pathway. Inhibition of AKT also increased levels of PRB. Previously, we demonstrated that a different AKT inhibitor, API-59CJ-OMe (EMD4Biosciences), also significantly increased PR levels in PRB23 Ishikawa cells [8]. Gu et al., [27], recently showed that inhibition of PI3K using LY294002 increased PR levels in Ishikawa cells. The influence of the AKT pathway on PR expression and function has not been studied in detail and the mechanisms by which this increase in PR protein occurs are currently being explored in our lab. Our current working hypothesis centers on the role of AKT in decreasing sensitivity to progesterone in endometrial cancer. Decreasing PR levels was the first piece of evidence supportive of our hypothesis. It was assumed that increased PR levels should enhance PR transcriptional function, however, data reveal that PR action is more complex, since not all progesterone-regulated genes were influenced by AKT inhibition. It is well established that, like other steroid hormone receptors, regulation of genes by PR involves numerous interactions with other transcription factors, DNA, coregulators, and chromatin remodeling complexes. PR action is gene specific in that different mechanisms are employed at different sites of the chromatin. Our microarray analysis identified PDK4 to be the most highly upregulated gene (more than 50-fold increase) with short-term progestin treatment. This was also a gene that was further increased with progestin when AKT was inhibited, indicating that overactive AKT in the Ishikawa cells dampens the progesterone action on this particular gene. It has been shown that PDK4 is also upregulated by glucocorticoids, through GR, in HepG2 cells [28]. They demonstrated that overexpression of PKBα abrogated the dexamethasone stimulation of the PDK4 promoter which supports our findings. Furthermore, they demonstrated that FOXO1, which is directly phosphorylated by AKT and exported to the cytoplasm, was required for the GR-mediated upregulation of PDK4. We have demonstrated a cooperation of PR and FOXO1 in regulating genes in endometrial stromal cells [29], [30], [31] and thus it is likely that PDK4 upregulation by progestins requires both PR and FOXO1 in endometrial cancer cells. Thus, when AKT is active, insufficient levels of FOXO1 are available to act in the nucleus and expression of PDK4 is dampened. It would be interesting to determine the significance of the PR and FOXO1 cooperation in the other progesterone-responsive genes that were influenced by AKT inhibition in endometrial cancer.

PDK4 is a kinase that inactivates the pyruvate dehydrogenase complex (PDC). The PDC generates acetyl-CoA, which is the precursor for fatty acid synthesis and energy production by the Krebs cycle. PDC activity is regulated by PDKs and PDC phosphatases to phosphorylate or dephosphorylate the E1a component of the complex. This regulation is important to control glucose and lipid metabolism. Upregulation of PDK4 by progestins and a further enhancement of this increase upon MK-2206 treatment suggests that progestins can act to inactivate the PDC and inhibit the conversion of pyruvate to acetyl-CoA. This inhibition of acetyl-CoA production may consequently lead to the utilization of alternate metabolic pathways such as glycolysis. Given the central role that PDK4 plays in regulating the choice between glycolysis and oxidative phosphorylation, PDK4 has been implicated in a number of different cancers. It has previously been shown that inhibition of PDK activity is sufficient to inhibit proliferation and induce apoptosis in lung cancer cells, suggesting that PDKs may drive tumorigenesis [32]. However, recent studies have also demonstrated that overexpression of PDK4 is sufficient to inhibit proliferation of breast cancer cells and that PDK4 expression is downregulated in a number of different cancer tissues [33]. The specific role that PDK4 plays in endometrial cancer remains to be deciphered.

Preclinical studies have demonstrated MK-2206 to effectively inhibit the AKT pathway as well as decrease proliferation and tumor growth of various cancer cell lines [25]. It has also been shown that the combination of MK-2206 and chemotherapeutic agents is more effective in inhibiting tumor growth [25]. MK-2206 along with a MEK inhibitor was demonstrated to have a synergistic effect on tumor growth and led to increased survival rates in mice bearing highly aggressive human lung tumors [34]. MK-2206 was able to restore sensitivity of resistant cells to sorafenib, a tyrosine kinase inhibitor, to induce apoptosis in hepatocellular carcinoma cells [35]. In combination with Gefitinib, a small molecule inhibitor of the EGFR tyrosine kinase, MK-2206 effectively promoted apoptosis in malignant glioma [17]. In addition to proliferation and apoptosis, it has been shown that MK-2206 inhibits metastasis of head and neck squamous cell carcinoma [18]. There are currently over 20 NCI-sponsored clinical phase I or II trials using MK-2206 for various malignancies. This compound, as a single agent, has been shown to be safe in humans and orally effective [21]. There is a phase II clinical trial currently underway utilizing MK-2206 for patients with PIK3CA mutation in recurrent or advanced endometrial carcinoma. MK-2206 is also being tested in combination with different chemotherapeutic regimens for various malignancies. The use of MK-2206, which has been shown to be safe and clinically effective in humans, could be considered in combination with progestin therapy for endometrial cancer patients who may be resistant to conventional therapy with progesterone. Furthermore, those patients that have endometrial tumors that are PTEN-negative, or carry mutations in the PIK3CA gene, could be considered for combinatorial MK-2206 and progestin therapy.

Progestin therapy has been used for decades for many women as an alternative to surgery for endometrial neoplasias. Studies have shown a 50–70% overall response rate for patients treated with high dose progestins as primary therapy. Close follow-up of patients treated with progestins is usually done, even in the responders because of the substantial rate of recurrence [36], [37], [38], [39], [40]. As risk factors and incidence of endometrial cancer are rising, the use of progestins will become more prevalent for prevention and treatment of this disease. Improving response rates to progestins will require a clearer understanding of the molecular mechanisms of progesterone receptor action and the pathways that influence its function.

We have demonstrated that inhibition of AKT not only increases PRB protein levels, but that it acts with progestin to decrease cell viability, increase apoptosis, and cause a decrease in tumor size. Our results indicate that inhibition of the AKT pathway could improve the efficacy of progestins in the treatment of endometrial cancer.
